# Sex-related differences in the effect of rotational thrombectomy for thrombus-containing lower limbs ischemic lesions

**DOI:** 10.1186/s12959-022-00437-4

**Published:** 2022-12-17

**Authors:** Jinyan Xu, Qingyuan Yu, Guanglang Zhu, Zhiqing Zhao, Yu Xiao, Junmin Bao, Liangxi Yuan

**Affiliations:** 1 Department of Vascular Surgery, Changhai Hospital, Navy Military Medical University, 168 Changhai Road, Shanghai, 200433 People’s Republic of China; 2 Department of Vascular Surgery, Qingpu Branch of Zhongshan Hospital, Affiliated to Fudan University, Shanghai, 201700 China

**Keywords:** Sex, Rotational, Thrombectomy, thrombus, Embolism, Lower-limb ischaemic

## Abstract

**Background:**

To assess the immediate effect and factors affecting the efficacy of rotational thrombectomy (RT) in patients with thrombus-containing lower-limb ischaemic lesions.

**Methods:**

Patients were retrospectively divided into two groups: RT and RT+ CDT (Catheter-directed thrombolysis). The RT group included patients in whom intraoperative thrombus aspiration was successful, while the RT + CDT group included patients in whom intraoperative thrombus aspiration was less effective and remedial CDT treatment was used. The primary outcome was the immediate effect of RT on thrombus-containing lower-limb ischaemic lesions.

**Results:**

From May 2015 to July 2021, 170 patients (113 men, 57 women; mean age, 74.0 years) with thrombus-containing lower-limb ischaemic lesions were treated in our centre. Of these patients, 113 received RT only, while 57 received RT + CDT. There were no significant intergroup differences in terms of age, disease duration, or comorbidities, but a higher proportion of male patients and higher preoperative plasma D-dimer levels (1.23 vs. 0.84; *p* = .017) was observed in the RT + CDT group. There were no significant intergroup differences in terms of diagnosis, lesion characteristics, lesion location, or lesion length. Multivariate logistic regression analysis revealed that male sex (odds ratio [OR], 2.65; 95% confidence interval [CI], 1.098–6.410; *p* = .030) and poor distal runoff (OR, 2.94; 95% CI, 1.439–5.988; *p* = .003) were associated with higher rates of additional CDT. Male patients also had a significantly longer onset time, more thrombotic occlusions, and a greater frequency of in-stent restenosis.

**Conclusions:**

RT alone or with CDT is a feasible primary treatment option for thrombus debulking. Sex significantly influences the effect of RT on thrombus-containing lower-limb ischaemic lesions.

## Introduction

Thrombus-containing ischaemic lesions in the lower limbs, caused by reduced arterial perfusion to a limb due to embolic migration or local thrombosis, are the most common condition observed in vascular emergencies [1]. Arterial occlusion caused by thrombosis or embolism, the most common cause of acute or subacute lower-limb ischaemia, may give rise to life-threatening complications [2]. Effective and suitable revascularization procedures should be performed in a timely manner to dredge thrombus-containing ischaemic lesions in the lower limbs, and adequate debulking is a rapid and effective method for managing thrombus formation.

The appropriate debulking method is selected according to the time of onset, nature of the lesion, general patient situation, and risk of thrombolytic bleeding. Catheter-directed thrombolysis (CDT) is traditionally used to treat lower-limb ischaemia, but this technique has clear limitations in patients at increased bleeding risk, especially those at risk of fatal haemorrhage [3–5].

Rotational thrombectomy (RT) is a safe and effective alternative treatment for patients with thrombus-containing lower-limb ischaemic lesions, demonstrating several advantages, such as the characteristics of endovascular treatment, the ability to rapidly remove thrombi, and a low risk of bleeding, which give RT good application prospects [6, 7]. However, the therapeutic effect of RT in some patients is ineffective, and further thrombolytic therapy is needed. It is unclear whether clinical factors such as lesion length, disease duration, or lesion nature are the key factors affecting the therapeutic effect of RT. Thus, this study aimed to investigate the followings: (1) the immediate effect of RT on thrombus-containing lower-limb ischaemic lesions, (2) risk factors for treatment outcomes of RT.

## Methods

### Patients and study design

This study was performed following the principles outlined in the Declaration of Helsinki and approved by the Changhai Hospital Medical Ethics Committee.

This single-centre retrospective study selected all patients who underwent RT treatment for thrombus-containing lower-limb ischaemic lesions in our centre between May 2015 and July 2021. The inclusion criterion was the presence of thrombus-containing lesions in the lower limbs without infrapopliteal thrombus involved. The exclusion criteria were thromboangiitis obliterans (TAO) or single infrapopliteal thrombus.

Patient demographics, comorbidities, laboratory examination results, lesion characteristics, and procedural details were also recorded. Comorbidities included hypertension, diabetes, smoking, coronary artery disease, atrial fibrillation, cerebrovascular disease, and chronic renal failure. Preoperative laboratory examinations included white blood cell (WBC) count and preoperative plasma D-dimer level.

### Procedure

The diagnosis of thrombus was established using angiography, the operator’s perception of the guidewire through the target lesion, and an intralesional angiogram. We determined whether the thrombus was fresh based on medical history, laboratory examination, and angiography findings. The RT device used in this study was a rotational system (Straub Medical, Wangs, Switzerland). When combined with atherosclerotic lesions, we usually selected a 3-mm-diameter balloon for the predilation of proximal lesions with a distal no-ballooning length of approximately 3 cm. The RT device was slowly moved through the lesions forwards and backwards with 3–4 passages, but only backwards passages were used in the iliac and distal popliteal arteries to reduce the risk of vessel perforation. The effect of thrombectomy was clearly confirmed by segmentation and intralesional angiography, and an additional two passages were used to treat any residual thrombi. Additional CDT was performed when we confirmed the presence of residual thrombus despite RT. After mechanical debulking, an appropriate balloon was selected to perform percutaneous transluminal angioplasty for lesions with > 30% residual stenosis. Distal runoff assessments before and after the intervention for the patients were performed together by two vascular surgeons who had extensive experience in vascular surgery. Patients were retrospectively divided into two groups: RT and RT + CDT. The RT group included patients in whom intraoperative thrombus aspiration was successful, while the RT + CDT group included patients in whom intraoperative thrombus aspiration was less effective and remedial CDT treatment was used. Intraoperative RT-related comorbidities included distal embolization, arterial perforation, and guidewire fractures.

### Outcomes

The primary outcomeon thrombus-was CDT use after RT therapy containing lower-limb ischaemic lesions.

### Definition

In our study, acute lower-limb ischaemia was defined as a decrease in limb arterial perfusion occurring within 2 weeks, subacute ischaemia was defined as a decrease in perfusion within 2 weeks to 3 months, and chronic ischaemia was defined as a decrease in perfusion within 3–6 months. Good distal runoff was defined as good superficial or deep femoral artery outflow for lesions involving the aortoiliac artery and at least two infrapopliteal arteries for lesions involving the femoropopliteal artery [8, 9]. Procedural success was defined as technical success and residual stenosis of ≤30%.

### Statistical analysis

Analyses were performed using SPSS software (version 26.0; SPSS, Chicago, IL, USA). Categorical data are presented as numbers and percentages. Nonnormally distributed data are expressed as medians and interquartile ranges. Intergroup differences were compared using the chi-squared test and Fisher’s exact test for categorical variables and Student’s t test for continuous variables. Statistical significance was set at *p* < .05. Factors identified through univariate analysis (*p* < .1) and other variables to be likely to have important prognostic value were tested in a multivariable logistic regression model of the RT effect. Odds ratios (ORs) and 95% confidence intervals (CIs) were estimated by using a logistic regression model. The stepwise regression method was used to screen independent variables. It performs partial F test on all independent variables during the screening process. Only variables smaller than the size of alpha value of 0.1 will be introduced into the equation. After that, the selected variables are tested one by one, and if the originally introduced variable becomes no longer significant due to the introduction of the latter variable, it will be eliminated.

## Results

### Baseline patient characteristics

The patients’ demographic and baseline characteristics are illustrated in Table 1. Overall, the mean patient age was 74.0 years, and 66.5% of them were men. More. than half of the patients had a medical history of hypertension and were in the acute stage of lower-limb ischaemia. A total of 113 patients received RT only (RT group), while 57 received a combination treatment of RT + CDT (RT + CDT group). Patients in the RT group were older and more likely to be female, while those in the RT + CDT group were more likely to be male. However, there were no significant intergroup differences in disease duration, hypertension, diabetes, smoking status, coronary artery disease, cerebrovascular disease, atrial fibrillation, chronic renal failure, WBC count, or preprocedural medications. The RT + CDT group had significantly higher plasma D-dimer levels (1.23 vs. 0.84; *p* = .017).

**Table 1:** Baseline clinical characteristics for RT and RT+CDT Groups

	Total	RT	RT + CDT	*P* value
Patients	170	113 (66.5%)	57 (33.5%)	
Median age	74.0 (65.8, 83.0)	76.0 (66.0, 84.0)	72.0 (63.5, 80.5)	.093
Sex				.005
Male	113(66.5%)	67 (59.3%)	46 (80.7%)	
Female	57(33.5%)	46 (40.7%)	11 (19.3%)	
Duration				.655
Acute	105 (61.8%)	68 (60.2%)	37 (64.9%)	
Subacute	27 (15.9%)	20 (17.7%)	7 (12.3%)	
Chronic	38 (22.4%)	25 (22.1%)	13 (22.8%)	
Comorbidities Hypertension	107 (62.9%)	74 (65.5%)	33 (57.9%)	.333
Diabetes	42 (24.7%)	26 (23.0%)	16 (28.1%)	.470
Smoking status	71 (41.8%)	50 (44.2%)	21 (36.8%)	.355
Coronary artery disease	34 (20.0%)	24 (21.2%)	10 (17.5%)	.570
Atrial fibrillation	58 (34.1%)	37 (32.7%)	21 (36.8%)	.595
Cerebrovascular disease	25 (14.7%)	17 (15.0%)	8 (14.0%)	.861
Chronic Renal failure	7 (4.1%)	5 (4.4%)	2 (3.5%)	.777
D-dimer	0.98 (0.55, 2.13)	0.84 (0.54, 1.57)	1.23 (0.74, 4.27)	.017
WBC	8.00(6.13, 11.11)	7.84 (5.96, 10.30)	8.82 (6.69, 12.43)	.073
Pre-procedural medication				.763
No	97 (57.1%)	65 (57.5%)	32 (56.1%)	
Antiplatelet only	55 (32.4%)	36 (31.9%)	19 (33.3%)	
Anticoagulant only	16 (9.4%)	10 (8.8%)	6 (10.5%)	
Anticoagulant+ Antiplatelet	2 (1.2%)	2 (1.8%)	0 (0.0%)	

*RT* Rotational thrombectomy, *CDT* Catheter-directed thrombolysis, *WBC* White blood cell Continuous data are presented as the means ± standard deviations; categorical data are given as the counts (percentage)

### Lesion characteristics and procedural details

The cause of ischaemia was established based on angiographic findings. Thrombus was diagnosed in 115 (67.6%) patients versus embolization in 55 (32.4%) patients. Overall, 110 (64.7%) patients had de novo lesions, and 60 (35.3%) had restenosis. The main target lesions were located in the aorto-iliac-femoral segment (*n* = 21), aortoiliac segment (*n* = 26), and femoropopliteal segment (*n* = 123). The two groups had similar lesion locations. Overall, 148 (87.1%) lesions were > 10 cm long.

Ninety-seven (57.1%) patients had good runoff vessels, whereas 73 (42.9%) had poor runoff vessels. Patients in the RT group displayed better distal runoff, whereas poor distal runoff was associated with CDT. There were no significant intergroup differences in diagnosis, lesion characteristics, lesion location, or lesion length (Table 2).

**Table 2:** Lesion characteristics and procedure details for RT and RT+CDT Groups

	Total	RT	RT + CDT	*P* value
Patients	170	113 (66.5%)	57 (33.5%)	
Diagnosis				.588
Thrombus	115(67.6%)	78 (69.0%)	37 (64.9%)	
Embolization	55(32.4%)	35 (31.0%)	20 (35.1%)	
Lesion characteristics				.968
De Novo	110(64.7%)	73 (64.6%)	37 (64.9%)	
Restenosis	60(35.3%)	40 (35.4%)	20 (35.1%)	
Lesion location				.805
Aorto-iliac-femoral artery	21(12.4%)	15 (13.3%)	6 (10.5%)	
Aorto-iliac artery	26(15.3%)	18 (15.9%)	8 (14.0%)	
Femo-popliteal artery	123(72.4%)	80 (70.8%)	43 (75.4%)	
Lesion length				.505
> 10 cm	148(87.1%)	97 (85.8%)	51 (89.5%)	
≤ 10 cm	22(12.9%)	16 (14.2%)	6 (10.5%)	
Distal runoff				.005
Good	97(57.1%)	73 (64.6%)	24 (42.1%)	
Poor	73(42.9%)	40 (35.4%)	33 (57.9%)	

Categorical data are given as the counts (percentage)

### Clinical and procedural outcomes

Although lesion types (diagnosis, characteristics, location, and length) did not differ significantly between the two groups, clinically significant differences were noted. Therefore, all variables were entered into a stepwise multivariate logistic regression model. For the reasons described above, the 14 variables described in Tables 1 and 2 were entered into a backwards stepwise multivariate logistic regression model. However, age, lesion length, lesion characteristics, lesion location, disease duration, atrial fibrillation, smoking status, and WBC were dropped in the stepwise analysis process. As a result, sex, distal runoff, and plasma D-dimer levels were independent factors for CDT after RT. Male sex was an independent risk factor for CDT after RT (OR, 2.65; 95% CI, 1.098–6.410; *p* = .030). Furthermore, poor distal runoff (OR, 2.94; 95% CI, 1.439–5.988; *p* = .003) was a predictor of CDT use.

Although plasma D-dimer levels (OR, 1.10; 95% CI, 1.003–1.202; *p* = .043) were risk factors for CDT, the OR of plasma D-dimer levels was not high (Table 3).

**Table 3:** Multivariate Analysis of Clinical and Procedural Predictors of CDT after RT

Endpoint	Variable	OR	95%CI	***P*** Value
**RT+ CDT**	Sex	2.65	1.098–6.410	.030
	Distal runoff	2.94	1.439–5.988	.003
	D-dimer	1.10	1.003–1.202	.043
	WBC	1.06	0.970–1.153	.205
	Age	1.02	0.985–1.057	.259

*OR* Odds ratio, *CI* Confidence intervals

As shown in Table 4, the mean age of the female patients was higher than that of the male patients (83.0 vs. 71.0 years, *p* < .001), while female patients had higher rates of atrial fibrillation (59.6% vs. 21.2%, *p* < .001), and male patients had higher smoking rates (61.1% vs. 3.5%, *p* < .001). Male patients had a significantly longer onset time, more thrombotic occlusions, and a greater frequency of in-stent restenosis lesions than female patients. Female patients had higher rates of a shorter onset time, embolic occlusions, and de novo lesions. Compared with male patients, more female patients received RT alone (80.7% vs. 59.3%, *p* = .005).

**Table 4:** Risk Factors and Baseline Characteristics Stratified by Sex

	Total	Male	Female	*P* Value
Patients	170	113 (66.5%)	57 (33.5%)	
RT only	113 (66.5%)	67 (59.3%)	46 (80.7%)	.005
Median age	74.0 (65.8, 83.0)	71.0 (63.0, 77.0)	83.0 (78.0, 86.5)	< .001
Atrial fibrillation	58 (34.1%)	24 (21.2%)	34 (59.6%)	< .001
Smoking status	71 (41.8%)	69 (61.1%)	2 (3.5%)	< .001
Duration				< .001
Acute	105 (61.8%)	58 (51.3%)	47 (82.5%)	
Subacute	27 (15.9%)	21 (18.6%)	6 (10.5%)	
Chronic	38 (22.4%)	34 (30.1%)	4 (7.0%)	
Diagnosis				.001
Thrombus	115 (67.6%)	86 (76.1%)	29 (50.9%)	
Embolization	55 (32.4%)	27 (23.9%)	28 (49.1%)	
Lesion characteristics				.001
De Novo	110 (64.7%)	63 (55.8%)	47 (82.5%)	
Restenosis	60 (35.3%)	50 (44.2%)	10 (17.5%)	

Data are shown as number (percentage) or median (interquartile range)

## Discussion

Endovascular thrombus debulking is the basis of endovascular treatment [6, 10], and percutaneous mechanical thrombectomy such as RT has emerged as an option in patients with acute or subacute lower-limb ischaemia [11]. When the RT efficacy was unsatisfactory, additional CDT was required to achieve thrombolysis [12]. In our study, we achieved primary success in 66% of the patients using RT only and in 34% of the patients using RT + CDT. The major findings of this study are as follow, (1) poor distal runoff was associated with CDT; (2) male sex was an independent predictor for adverse outcomes after RT.

Duc et al. [13] concluded that mechanical debulking with RT effectively treats acute, subacute, and even chronic thrombus lesions and is equally effective in the treatment of short- or long-segment thrombi. This is consistent with our finding that the RT system could be used to re-establish peripheral arterial flow regardless of lesion duration or length. Liang et al. [14] showed that percutaneous mechanical thrombectomy had the highest patency rate for acute arterial embolism, followed by acute arterial thrombosis, but the lowest rate for stent/graft thrombosis. Nevertheless, in our study, primary occlusion (both embolism and thrombosis) and stent/graft thrombosis had similar post-RT outcomes; we believe that the different outcomes may have been due to differences in the percutaneous mechanical thrombectomy methods. Previous studies reported poor results with rotational catheters for acute iliac artery occlusion, which may be due to the relative curvature of the iliac artery, while the rotational catheter lacks directionality and cannot reach the large curved side of arterial tortuosity. Another reason is that the diameter of the iliac artery is comparatively large, so the rotational catheter can reach only relatively smaller thrombi [15]. However, the use of the forwards and backwards passage methods in this study revealed no statistically significant differences in the effect of RT by lesion location.

Analyses of the factors for the poor effect of RT treatment are lacking [14, 16, 17], and our multivariate analysis revealed that only sex and distal runoff contributed to RT efficacy. In our study, the poor runoff group had unsatisfactory RT outcomes and required additional CDT. The rate of poor runoff in RT + CDT group was much higher than in RT group, which may be related to complex lesions in RT + CDT group.

Complicated lesion may increase the difficulty of thrombus aspiration. An infrapopliteal lesion is characterized by a small diameter and low outflow. When the RT catheter fills the infrapopliteal vessel, the vessel wall may embed in the lateral window of the catheter tips [18], and we use RT more conservatively to avoid the risk of perforation for infrapopliteal vessels.

Previous studies reported higher perioperative mortality and complication rates in females than in males after peripheral arterial surgery [19]. Female patients benefit less than male patients because of the greater mean age at onset, more advanced ischaemic phase, and lower target vessel quality [20]. Contrary to previous findings, in our study, male sex was another key factor influencing RT efficacy. Our data showed that female patients had shorter onset times, more atrial fibrillation, more de novo lesions, and higher rates of embolization, which suggested a higher proportion of cardiogenic emboli in female patients, although some cases of acute thrombosis could not be excluded. However, male patients had higher rates of thrombosis, more chronic lesions, and more restenotic lesions, all suggesting that males were more likely to develop thrombosis on the basis of atherosclerosis. Arteriosclerotic thrombi are mainly composed of old fibrin and platelets, whereas cardiogenic thrombi are primarily composed of young fibrin and erythrocytes [21, 22]. Erythrocyte-rich clots have higher variability and lower stiffness, whereas fibrin-rich clots are more likely to have greater thrombus stiffness and frictional forces, resulting in tighter and more stable thrombi [23–25]. This difference implies that cardiogenic thrombi are less resistant to mechanical rupture. A notably higher density of fibrin within clots was also demonstrated among smokers [26], and more than half of the men versus a minority of women in our study had a history of smoking.

RT is better for relatively fresh thrombus removal, whereas mechanical debulking is not always ideal in cases of endothelial hyperplasia and old thrombi. Even without contacting the catheter, a fresh thrombus can be aspirated into the catheter due to fragmentation caused by the negative pressure created by the catheter. However, a relatively old thrombus must make contact with the catheter to achieve debulking; otherwise, incomplete debulking can result in distal dislodgement of the residual thrombus and embolization of the distal arteries. In our study, a greater proportion of male versus female patients had chronic and restenosis lesions. This may explain the poor outcomes of the rotational system in men. This finding may have guiding significance for clinical practice: if male patients have a history of smoking with subacute or chronic thrombus, they are more likely to need RT combined with CDT treatment. This analysis has some major limitations. First, its retrospective nonrandomized design may have led to mistakes or inadequate data collection. Additionally, as this was a single-centre study, it included a limited number of patients and inherent limitations. These outcomes require confirmation in a larger study.

## Conclusion

RT alone or with CDT is a feasible primary treatment option for thrombus debulking to re-establish flow. Sex significantly influences the effect of RT on thrombus-containing lower-limb ischaemic lesions.

## Data Availability

The datasets generated and analyzed during the current study are not publicly available, as the experimental data are related to other experiments that are progressing but are available from the corresponding author upon reasonable request.

## Abbreviations

RT: Rotational thrombectomy
CDT: Catheter-directed thrombolysis
TAO: Thromboangiitis obliterans
WBC: White blood cell
OR: Odds ratio
CI: Confidence interval
